# TUDCA and 4-PBA in Preclinical Models of Beta-Cell Secretory Failure: A Systematic Review and Bayesian Meta-Analysis

**DOI:** 10.3390/ijms27167267

**Published:** 2026-08-14

**Authors:** Arnulfo Ramos-Jiménez, Mariazel Rubio-Valles, Jaime Guereca-Arvizuo, Javier A. Ramos-Hernández, Everardo González-Rodríguez, Verónica Moreno-Brito, Marco A. Juárez-Oropeza

**Affiliations:** 1 Institute of Biomedical Sciences, Autonomous University of Ciudad Juárez, Ciudad Juárez Campus, Chihuahua 32310, Mexico; jaime.guereca@uacj.mx; 2 Faculty of Physical Culture Sciences, Autonomous University of Chihuahua, University Circuit, Campus II, Chihuahua 31125, Mexico; p305510@uach.mx; 3 Faculty of Medicine, Autonomous University of Nuevo León, Monterrey 64460, Mexico; javier.ramoshrn@uanl.edu.mx; 4 Faculty of Medicine and Biomedical Sciences, Autonomous University of Chihuahua, University Circuit, Campus II, Chihuahua 31109, Mexico; evegonzal@uach.mx (E.G.-R.); vmoreno@uach.mx (V.M.-B.); 5 Department of Biochemistry, School of Medicine, National Autonomous University of Mexico, Mexico City 04510, Mexico; majo_ya@yahoo.com.mx

**Keywords:** beta-cell, endoplasmic reticulum stress, tauroursodeoxycholic acid, 4-phenylbutyrate, glucose-stimulated insulin secretion, unfolded protein response, type 2 diabetes mellitus, Bayesian meta-analysis, chemical chaperone

## Abstract

The progressive failure of pancreatic beta-cells under chronic glucolipotoxicity drives the pathogenesis of type 2 diabetes mellitus (T2DM). This metabolic stress overwhelms the folding capacity of the endoplasmic reticulum (ER), hyperactivates the unfolded protein response (UPR), engages terminal pro-apoptotic signaling through C/EBP-homologous protein (CHOP), and promotes beta-cell dedifferentiation. In this systematic review and meta-analysis, registered with PROSPERO (CRD420261370436), we evaluated the preclinical efficacy of the low-molecular-weight chemical chaperones tauroursodeoxycholic acid (TUDCA) and 4-phenylbutyrate (4-PBA) in preserving beta-cell exocytotic identity and mitigating ER stress. Following PRISMA 2020 guidelines, a systematic search of PubMed, Scopus, and Web of Science (January 2016–May 2026) identified four eligible experimental studies. Preclinical models (INS-1 and βTC-6 cell lines, Wistar rats, and C57BL/6 mice) exposed to a high-fat diet (HFD), a high-fat/high-fructose diet (HFHFD), cholesterol loading, or protein restriction followed by high-fat feeding showed impaired or dysregulated glucose-stimulated insulin secretion (GSIS) and upregulated ER-stress markers. Co-administration of TUDCA or 4-PBA moved secretory output toward the healthy-control phenotype in every model and reduced pro-apoptotic markers in the three models in which they were measured. A hierarchical Bayesian random-effects meta-analysis of the between-arm GSIS restoration ratio at stimulatory glucose yielded a pooled ratio of 1.85 (95% credible interval [CrI] 1.38 to 2.43), with the entire credible mass above the null (posterior probability of benefit 0.996). This estimate was stable across nine prior specifications for the between-study standard deviation and in every leave-one-out analysis, including exclusion of the single hypersecretion model (1.98, 95% CrI 1.09 to 3.18). Between-study variance was small but weakly identified from only four studies and is reported as exploratory. Pooling instead on the registered within-arm stimulation-index scale, a change in metric declared as a protocol deviation, gave 1.46 (95% CrI 0.73 to 2.58) with substantial heterogeneity (I^2^ = 89.3%), so the evidence supports restoration of absolute glucose-stimulated insulin output rather than of fold glucose responsiveness. Because no source report documents blinding of outcome assessment, the pooled estimate should be read as an upper bound. In conclusion, TUDCA and 4-PBA act as chemical chaperones that alleviate ER stress and may prevent terminal UPR activation and preserve the beta-cell exocytotic machinery, positioning them as candidate disease-modifying agents that merit confirmatory clinical evaluation.

## 1. Introduction

The relentless global rise in type 2 diabetes mellitus (T2DM) represents one of the most severe public-health and socioeconomic challenges of modern medicine, characterized by peripheral insulin resistance and progressive pancreatic beta-cell functional failure [1]. Initially, the endocrine pancreas compensates for diminished peripheral insulin sensitivity by hyperactivating its synthetic and exocytotic machinery, maintaining normoglycemia at the expense of chronic hyperinsulinemia [2]. However, persistent exposure to elevated blood glucose and circulating non-esterified free fatty acids, a pathological state termed “glucolipotoxicity,” imposes an unsustainable synthetic burden on beta-cells [3]. This burden is associated with ER redox imbalance, delayed ER export, accumulation and misfolding of proinsulin, and beta-cell dysfunction [4].

The ER is the primary site for structural folding, disulfide-bond formation, and quaternary assembly of secretable proteins, and in the beta-cell, the folding and disulfide maturation of proinsulin depend directly on the redox state of the ER lumen [4]. Pancreatic beta-cells are highly specialized for insulin production, but chronic glucolipotoxicity increases the misfolded-protein burden in the ER, elevating BiP/GRP78 and other chaperones and robustly activating all three branches of the UPR. Initially, this response aims to reduce protein load and enhance folding; however, with persistent glucolipotoxicity, the UPR shifts toward pro-apoptotic outputs (CHOP, JNK, mitochondrial apoptosis), contributing to beta-cell failure [5,6,7].

In its physiological phase, the UPR initiates an adaptive feedback loop to restore proteostasis. Following dissociation from BiP, PERK oligomerizes and autophosphorylates, phosphorylating the α-subunit of eukaryotic initiation factor 2 (eIF2α) and inducing a transient global attenuation of translation [1]. Concurrently, activated IRE1α displays endoribonuclease activity, processing Xbp1 mRNA to produce splicing-active XBP-1s, which transcriptionally upregulates ER chaperones and ER-associated degradation components [1]. Simultaneously, ATF6 translocates to the Golgi apparatus for proteolytic cleavage, releasing its active cytosolic domain to act as a pro-survival transcription factor [1].

When ER stress is chronic, severe, and unresolved, the UPR transitions from a pro-survival program to a terminal pro-apoptotic cascade [5]. Prolonged phosphorylation of eIF2α selectively promotes translation of activating transcription factor 4 (ATF4), which upregulates transcription of Ddit3, encoding C/EBP-homologous protein (CHOP) [1]. Nuclear CHOP accumulation suppresses anti-apoptotic Bcl2 and activates pro-apoptotic Bax, Bim, and p53, triggering mitochondrial outer-membrane permeabilization, cytochrome-c release, and caspase-3-dependent apoptosis; in the beta-cell, this PERK–eIF2α-dependent route is the principal ER-stress pathway implicated in type 2 diabetes mellitus [3,5]. Crucially, before cell death is finalized, the loss of proteostasis drives beta-cells into functional silence and epigenetic dedifferentiation, downregulating identity genes such as PDX-1, MAFA, and NKX6.1 and silencing the calcium-dependent exocytotic machinery, thereby abolishing glucose-stimulated insulin secretion (GSIS) [6].

To preserve exocytotic identity, pharmacological strategies utilizing low-molecular-weight chemical chaperones have gained significant interest [7]. TUDCA and 4-PBA act as molecular scaffolds, stabilizing protein conformation, accelerating folding kinetics, and reducing the burden of ER-misfolded proteins. Although preclinical evidence suggests these agents are beneficial, the literature is characterized by small-sample studies and methodological noise, hindering quantitative synthesis. To address these limitations, we conducted a PRISMA-compliant systematic review and a hierarchical Bayesian meta-analysis of preclinical studies assessing the efficacy of TUDCA and 4-PBA in restoring GSIS and mitigating terminal ER stress under metabolic glucolipotoxicity (PROSPERO CRD420261370436).

## 2. Materials and Methods

### 2.1. Protocol Registration and Search Strategy

This systematic review was conducted in accordance with the Preferred Reporting Items for Systematic Reviews and Meta-Analyses (PRISMA 2020) guidelines [8] and drew on the methodological standards of the Cochrane Collaboration. The protocol was registered prospectively in PROSPERO (CRD420261370436). Deviations from that registration are declared in Section 2.6.

A systematic search was conducted in PubMed (MEDLINE), Scopus, and the Web of Science Core Collection. Although the registered protocol additionally listed EBSCO as a candidate database, EBSCO did not provide indexing coverage beyond the three core databases for the target literature and was therefore not searched; this deviation is declared in Section 2.6. The Boolean search syntax was: (“pancreatic beta cell” OR “beta-cell” OR “islet of Langerhans”) AND (“endoplasmic reticulum stress” OR “ER stress” OR “glucolipotoxicity” OR “lipotoxicity”) AND (“chemical chaperone” OR “TUDCA” OR “4-PBA”) AND (“glucose-stimulated insulin secretion” OR “GSIS” OR “CHOP”). The registered protocol specified an end date (search current through 2026) without a fixed start date; an a priori decision, taken before screening and not revised after the results were known, restricted the analytic window to January 2016–May 2026 in order to focus the quantitative synthesis on contemporary studies that employed standardized glucolipotoxic protocols and reported quantitative insulin-secretion data at both basal and stimulatory glucose. The principal consequence of this restriction is that the quantitative synthesis rests on four studies, which in turn limits the precision with which between-study variance can be estimated; that limitation is quantified directly in the prior-sensitivity analysis (Section 2.5 and Section 3.4) and discussed in the Limitations (Section 4.6).

### 2.2. Eligibility Criteria (PICOS Framework)

Predefined PICOS criteria were applied for study selection:

• Population (models): preclinical in vivo rodent models, primary isolated islets ex vivo (human or murine), or validated immortalized pancreatic beta-cell lines (e.g., INS-1, INS-1E, MIN6, βTC-6).
• Intervention: pharmacological administration of low-molecular-weight chemical chaperones (TUDCA and/or 4-PBA) under standardized metabolic (lipotoxic or glucolipotoxic) stress.
• Comparator: vehicle-treated controls exposed to the identical metabolic stress (the “stressor-only” arm).
• Outcomes: the registered primary outcome was the GSIS stimulation index (the ratio of insulin secreted under stimulatory high-glucose vs. basal low-glucose conditions). For quantitative pooling, this was operationalized as the between-arm GSIS restoration ratio at stimulatory glucose, a deviation from the registered protocol that is defined in Section 2.4, declared and justified in Section 2.6, and examined empirically in Section 3.4. The registered secondary outcome was the relative expression of the terminal pro-apoptotic marker CHOP. Three further readouts—the ER chaperone BiP/GRP78, the ER-membrane glycoprotein Wolfram syndrome 1 (WFS1), and cleaved caspase-3—were added after registration and are reported throughout as additional, mechanistically motivated outcomes rather than as registered ones. They were added because all three lie on the same PERK–ATF4–CHOP axis as the registered marker and were reported by most eligible studies, so that omitting them would have discarded directly relevant mechanistic evidence; they were not used to select studies, to define eligibility, or to compute any pooled effect size. Availability of ER-stress markers was never an eligibility requirement: eligibility was determined by the population, the intervention, the comparator, and the availability of insulin-secretion data.
• Study design: original experimental basic-science research articles published in peer-reviewed indexed journals in English. Narrative reviews, observational studies, and trials using non-physiological chemical-only ER stressors (e.g., thapsigargin or tunicamycin without a lipid/glucose metabolic component) were excluded.

### 2.3. Study Selection, Data Extraction, and Risk-of-Bias Assessment

Titles and abstracts, and then the full texts of the records retained, were screened and read independently by two reviewers against the PICOS criteria, and disagreements were resolved by consensus. Two reviewers independently extracted data and resolved discrepancies by consensus. Extracted variables included study design, biological model, metabolic stressor, chaperone dosing, primary GSIS outcomes, secondary CHOP and BiP expression, and systemic metabolic parameters. Dosing regimens, group sizes, glucose concentrations, stressor protocols, and the dispersion measure reported by each source (standard error of the mean or standard deviation) were taken directly from the text, tables, and figure legends of the primary reports and are fully verifiable. For studies presenting the insulin-secretion outcome graphically, numerical values were extracted from the source figures using WebPlotDigitizer (version 4.6), an instrument whose inter-coder reliability and validity for extracting graphed data have been formally evaluated [9]. Every extracted value was subsequently re-read from the source figure by a second reader working independently of the original extraction, and the two sets were reconciled before any model was fitted; across the eight primary values entering the meta-analysis, the mean absolute relative difference between the two readings was 2.8% (maximum 5.3%), and consensus values were retained. Because this residual error is not captured by the standard errors reported in the source figures, its effect on the pooled estimate was bounded explicitly by refitting the model with additional per-arm relative error added in quadrature (Section 2.5). The corresponding authors of the four primary reports were not contacted, and the underlying numerical datasets were not requested from them; the synthesis rests exclusively on the published full texts of those reports and on the values digitized from their figures, so extraction error was addressed analytically rather than by recourse to the original data. The methodological quality and risk of bias of the included studies were assessed using the SYRCLE risk-of-bias tool for animal studies [10], which covers selection, performance, detection, attrition, and reporting biases.

### 2.4. Hierarchical Bayesian Random-Effects Meta-Analysis

**Figure 1:**
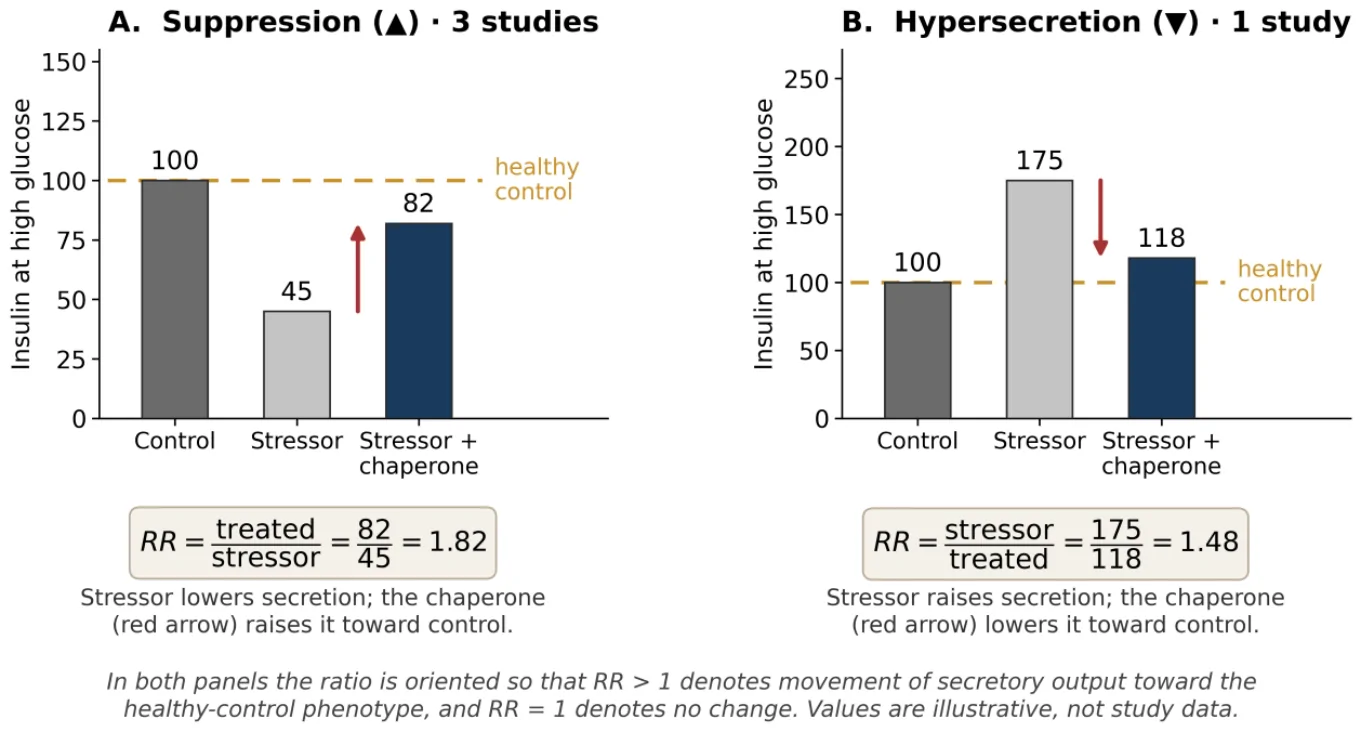
Definition of the GSIS restoration ratio in the two secretory phenotypes represented among the included studies. (**A**) In the three suppression models, the metabolic stressor lowers insulin secretion at stimulatory glucose, and the chemical chaperone raises it, so the ratio is computed as treated ÷ stressor. (**B**) In the single hypersecretion model, the stressor raises secretion above the healthy-control level and the chaperone lowers it, so the ratio is computed as stressor ÷ treated. In both cases, the ratio is oriented so that values above 1 denote movement of secretory output toward the healthy-control phenotype and a value of 1 denotes no change. The numbers shown are illustrative and are not study data.

To model the true biological variance (τ^2^) and to mitigate the small-sample bias common in basic science [11], a hierarchical Bayesian random-effects meta-analysis was performed on the log-transformed GSIS restoration ratio. For each study, this restoration ratio was operationalized as the between-arm ratio of insulin secretion measured at stimulatory glucose, computed as the treated-to-stressor ratio in the three models in which the metabolic stressor suppressed secretion and as the stressor-to-treated ratio in the single model in which it produced hypersecretion, so that values above unity uniformly denote normalization of secretory output toward the healthy-control phenotype; Figure 1 illustrates both cases and shows how they map onto a single scale. This between-arm metric is not identical to the within-arm GSIS stimulation index named in the registered protocol. The two are related exactly by RR = (SI_treated/SI_stressor) × (B_treated/B_stressor), where SI denotes the within-arm stimulation index and B the insulin secreted at basal glucose in the same arm. The between-arm ratio therefore equals the ratio of stimulation indices only when basal secretion is unaffected by the chaperone and departs from it in exact proportion to any chaperone-induced change in basal output; the empirical size and direction of that departure are quantified for every study in Section 3.4 and discussed in Section 4.3. Let y_i_ denote the estimated log effect size in study i and s_i_^2^ the within-study sampling variance, obtained by the delta method from the reported group means and their standard errors. The model was specified as.

*y_i_* ~ *Normal*(*θ_i_*, *s_i_*^2^); *θ_i_* ~ *Normal*(*μ*, *τ*^2^)

Weakly informative priors were used: a normal prior on the global mean effect, μ ~ Normal(0, 10^2^), and a half-Cauchy prior on the between-study standard deviation, τ ~ Half-Cauchy(0, 1) with τ > 0, in line with current recommendations for Bayesian random-effects meta-analyses of few small studies [12,13,14]. Because a heterogeneity parameter estimated from four studies is only weakly identified by the data, the dependence of the posterior on this choice was not assumed to be negligible but was quantified directly across eight alternative specifications (Section 2.5). The joint posterior was computed exactly. Because the marginal likelihood of this normal–normal hierarchical model is available in closed form conditional on τ, every posterior summary reported in this article was obtained by deterministic numerical integration over τ on a dense grid, so the reported quantities carry no Monte-Carlo error. As an independent cross-check, the posterior was also estimated by Markov-chain Monte Carlo using the No-U-Turn Sampler (NUTS) implemented in PyMC, with a non-centered parameterization to ensure stable geometry. Four independent chains were run with 5000 tuning iterations and 20,000 retained draws each (80,000 posterior samples in total), a target acceptance probability of 0.99, and a fixed random seed for reproducibility. That chain converged (Gelman–Rubin R-hat ≤ 1.001; effective sample sizes above 12,000 for all reported parameters) and agreed with the exact posterior to within 0.3% for the posterior mean and 0.9% for the limits of the credible interval. Nineteen divergent transitions occurred after tuning; they are reported for completeness and do not affect any reported quantity, since all reported quantities come from the exact integration rather than from the sampler. Cochran’s Q and I^2^ are reported alongside τ^2^ as prior-free descriptors of heterogeneity [15]. Because only four studies met the eligibility criteria, formal assessment of small-study effects and publication bias (e.g., funnel-plot asymmetry or Egger’s regression) was not undertaken, as such tests are underpowered and unreliable when fewer than approximately ten studies are available [16].

### 2.5. Sensitivity and Robustness Analyses

Five sets of sensitivity analyses were specified, all using the same data, likelihood, and software as the primary analysis, with only the stated element varied. First, leave-one-out analyses removed each study in turn; the analysis omitting the single hypersecretion model was designated in advance as the principal robustness check, because that model is the only one whose untreated phenotype is directionally opposite to the remaining three. Second, the prior on the between-study standard deviation was varied across eight alternatives spanning a wide range of prior mass near zero and in the tail—Half-Cauchy(0, 0.5), Half-Cauchy(0, 0.25), Half-Normal(0, 0.5), Half-Normal(0, 0.25), Exponential with mean 1 and with mean 0.3, Uniform(0, 2), and a flat reference prior on τ—and both the pooled effect and τ^2^ were re-estimated under each, in the full set and in the suppression-only subset. Third, additional per-arm relative digitization error of 2%, 5%, 10%, and 15% was added in quadrature to the within-study standard errors, so that the sensitivity of the pooled estimate to extraction error could be read directly rather than asserted.

Fourth, the operationalized metric was compared with the registered within-arm stimulation index in every study, using the basal-glucose secretion values reported in the same source figures (ref. [17], Figure 5A; ref. [18], Figure 8A, left block; ref. [19], Figure 4K, basal insulin secretion (“BIS”) bars; ref. [20], Figure 3K, 2.8 mM block). The same hierarchical model was then re-fitted to the four per-study stimulation-index ratios, so that the pooled estimate on the registered scale could be compared directly with the pooled estimate on the operationalized scale rather than only study by study; in that re-fit, the basal ratio is treated as known, and the within-study standard errors are left unchanged, an assumption that narrows rather than widens the resulting interval. Cochran’s Q and I^2^ were computed on both scales. Fifth, two data-provenance analyses were run: one entering the dispersion of the study that reports standard deviations of three to five independent experiments [19] as a standard error without converting it, as in the original submission, and one retaining the originally submitted reading of the treated arm of [18]. All results are reported in Section 3.4.

### 2.6. Deviations from the Registered Protocol

Four deviations from the PROSPERO registration are declared. First, the registered protocol listed EBSCO as a candidate database; it was not searched, for the reason given in Section 2.1. Second, the effect-size metric used for quantitative pooling is the between-arm GSIS restoration ratio at stimulatory glucose rather than the registered within-arm GSIS stimulation index. This operationalization was adopted because the stimulation index is a ratio of two quantities that chronic glucolipotoxicity alters in the same direction but to different degrees, so that a preserved or even elevated stimulation index can coexist with a collapse of absolute secretory capacity; the between-arm ratio at stimulatory glucose measures the restoration of secretory capacity directly and was reported by all four eligible studies on a common footing. The direction and magnitude of the discrepancy between the two metrics are not assumed to be negligible: they are quantified study by study in Section 3.4 and discussed in Section 4.3. Third, BiP/GRP78, WFS1, and cleaved caspase-3 are reported alongside the registered secondary outcome CHOP as additional, post-registration mechanistic outcomes (Section 2.2). Fourth, the registered protocol specified an end date for the search but no start date; the analytic window was restricted to January 2016–May 2026 before screening, for the reason given in Section 2.1. This restriction is the proximate reason that only four studies were eligible, and therefore the proximate reason that the between-study variance is weakly identified; it is declared here as a deviation because the registration does not contain it. No other aspect of the registered protocol was changed, and no deviation was introduced after the pooled estimate had been inspected.

## 3. Results

### 3.1. Study Selection

**Figure 2:**
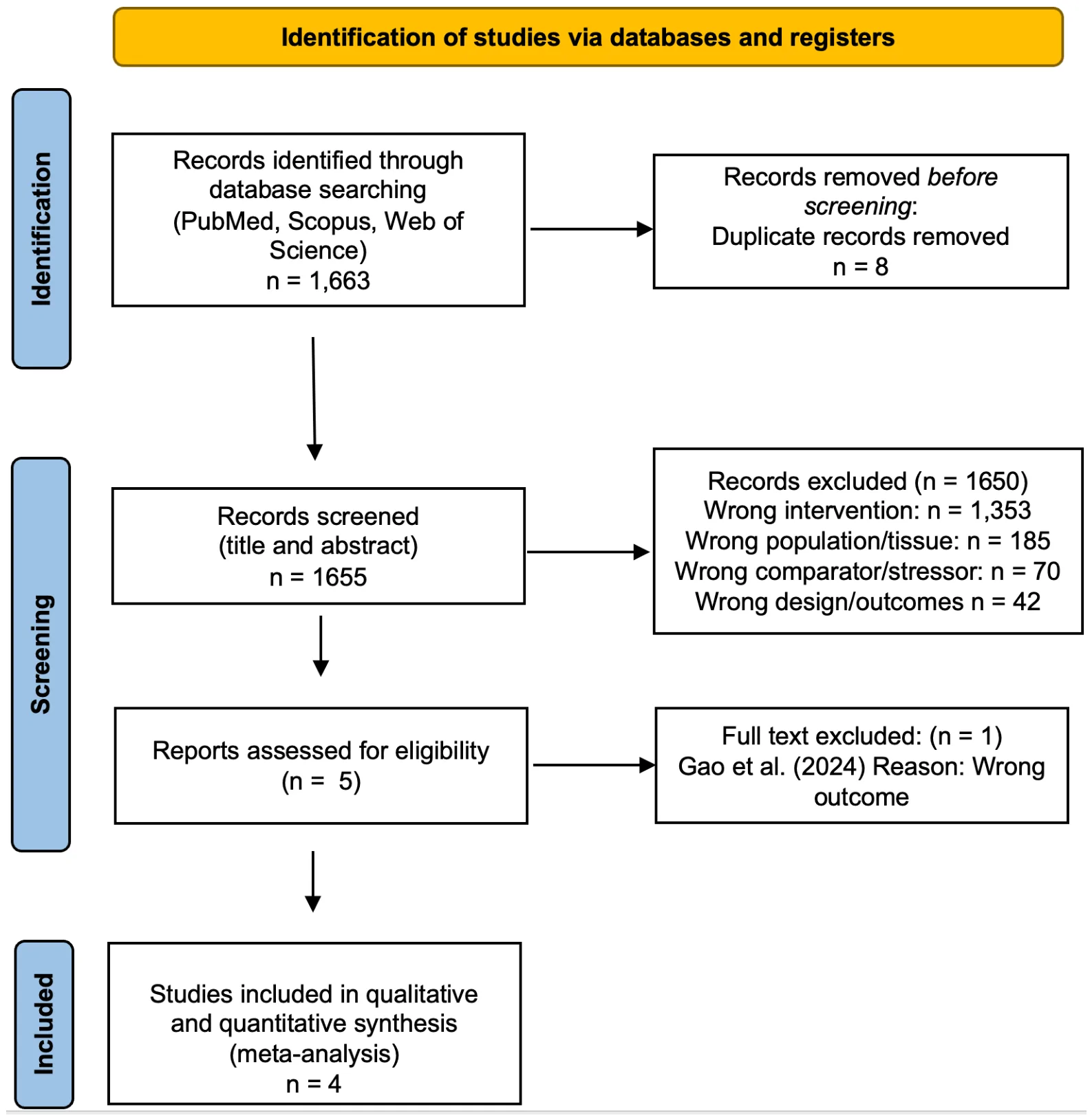
PRISMA 2020 flow diagram of the study-selection process. Record counts are reported as identified, screened, excluded with reasons, assessed at full text, and finally included. The single report excluded at full text was excluded because its primary outcome was glucagon-like peptide-1 (GLP-1) receptor signaling rather than glucose-stimulated insulin secretion or CHOP mitigation as specified in the PICOS criteria.

The systematic search of PubMed, Scopus, and Web of Science (January 2016–May 2026) returned 1663 records. After removal of eight duplicates, 1655 records were screened by title and abstract, of which 1650 were excluded against the PICOS criteria (1353 for wrong intervention, 185 for wrong population or tissue, 70 for wrong comparator or stressor, and 42 for wrong study design or outcomes). Five full-text articles were assessed for eligibility; one was excluded because its primary outcome concerned GLP-1-receptor signaling rather than the GSIS stimulation index or CHOP mitigation. Consequently, four preclinical experimental studies were included in the qualitative and quantitative syntheses [17,18,19,20]. The selection workflow is summarized in the PRISMA flow diagram (Figure 2).

### 3.2. Qualitative Synthesis and Risk of Bias

Application of the SYRCLE tool rated every domain as either low or unclear in the four included studies; the instrument has no intermediate category, and no domain was rated high. Selection bias was judged low because the in vivo studies reported random allocation to diet and treatment arms. Attrition bias was judged low, with complete datasets reported for the primary variables. Reporting bias was rated unclear rather than low: none of the four reports gives the primary insulin-secretion values numerically, so those values had to be read from the published figures (Section 2.3), which is itself an incompleteness of reporting. Detection bias was rated unclear in all four studies because blinding of outcome assessors during the insulin-secretion assays and Western blot densitometry is not documented in any of the source reports; this is a feature of the primary preclinical literature rather than of the present synthesis, and its implications for the interpretation and clinical generalizability of the pooled estimate are examined in Section 4.6. The study characteristics and risk-of-bias judgments are summarized in Table 1. The models ranged from in vitro immortalized cell lines (INS-1, βTC-6) to ex vivo isolated islets from in vivo rodents fed high-fat or high-fat/high-fructose diets [17,18]. One caveat applies to the instrument itself: SYRCLE was developed for animal studies, and the cholesterol model [19] is an in vitro study in immortalized cell lines. Its domains were assessed by analogy, and its risk-of-bias row in Table 1 should be read with that in mind.

### 3.3. Primary Outcome: Quantitative Restoration of Glucose-Stimulated Insulin Secretion

Chronic overnutrition and lipotoxic insults impaired or dysregulated insulin secretion at stimulatory glucose across dietary and lipotoxic models. In rodents fed a high-fat diet (HFD) [17] or a high-fat/high-fructose diet (HFHFD) [18], islets exhibited severe impairment of exocytotic capacity in response to high-glucose stimulation (16.7 mM). The same defect was reproduced in vitro after cholesterol loading of βTC-6 cells, in which insulin secretion was quantified, with concordant induction of CHOP and cleaved caspase-3 in both βTC-6 and INS-1 cells [19]. By contrast, in mice subjected to protein restriction followed by HFD, the islets developed glucose-stimulated insulin hypersecretion, which TUDCA normalized [20]. The per-study dosing regimens, high-glucose GSIS values, and resulting effect sizes are summarized in Table 2.

**Table 3:** Sensitivity and robustness analyses of the pooled GSIS restoration ratio. All models use the same data, likelihood, priors on μ, and software as the primary analysis; only the element named in each row is varied. CrI, credible interval; P (benefit), posterior probability that the restoration ratio exceeds 1; k, number of studies. Posterior summaries in this table were obtained by exact numerical integration over τ, which is available in closed form for this model; the primary-model row reproduces the estimate reported in Section 3.3, and values may differ from a sampler-based estimate in the last reported digit through Monte-Carlo error alone.

Analysis	k	Pooled Restoration Ratio (Posterior Mean)	95% CrI	τ^2^ (Median)	P (Benefit)
Primary model—τ ~ Half-Cauchy(0, 1)	4	1.85	1.38 to 2.43	0.019	0.996
Registered metric—pooled on the within-arm stimulation-index scale	4	1.46	0.73 to 2.58	0.196	0.894
Registered metric—stimulation-index scale, suppression-only	3	1.85	0.52 to 3.97	0.308	0.852
Leave-one-out—excluding the hypersecretion model [20] (=4-PBA only)	3	1.98	1.09 to 3.18	0.045	0.981
Leave-one-out—excluding Binayi et al. [17]	3	1.79	1.15 to 2.55	0.017	0.986
Leave-one-out—excluding Izadi et al. [18]	3	2.02	1.21 to 3.05	0.026	0.988
Leave-one-out—excluding Kong et al. [19]	3	1.96	1.02 to 3.24	0.056	0.977
Prior—τ ~ Half-Cauchy(0, 0.5)	4	1.84	1.43 to 2.34	0.015	0.998
Prior—τ ~ Half-Cauchy(0, 0.25)	4	1.84	1.49 to 2.25	0.010	0.999
Prior—τ ~ Half-Normal(0, 0.5)	4	1.84	1.42 to 2.35	0.016	0.999
Prior—τ ~ Half-Normal(0, 0.25)	4	1.84	1.49 to 2.24	0.011	1.000
Prior—τ ~ Exponential (mean 1)	4	1.85	1.40 to 2.39	0.016	0.997
Prior—τ ~ Exponential (mean 0.3)	4	1.84	1.49 to 2.25	0.010	0.999
Prior—τ ~ Uniform(0, 2)	4	1.86	1.32 to 2.54	0.022	0.994
Prior—flat reference prior on τ	4	1.89	1.30 to 2.57	0.022	0.992
Prior—Uniform(0, 2), suppression-only subset	3	1.97	0.93 to 3.70	0.063	0.969
Prior—flat reference prior on τ, suppression-only subset	3	3.27	0.66 to 5.18	0.073	0.951
Digitization error—+2% per arm, in quadrature	4	1.85	1.37 to 2.43	0.019	0.996
Digitization error—+5% per arm, in quadrature	4	1.85	1.36 to 2.45	0.018	0.996
Digitization error—+10% per arm, in quadrature	4	1.86	1.32 to 2.53	0.019	0.995
Digitization error—+15% per arm, in quadrature	4	1.87	1.25 to 2.68	0.024	0.993
Data provenance—Izadi et al. [18] treated arm at the originally submitted reading (190)	4	1.87	1.43 to 2.40	0.014	0.997
Data provenance—Kong et al. [19] dispersion entered as SEM, not rescaled from SD	4	1.85	1.34 to 2.49	0.023	0.996

**Figure 3:**
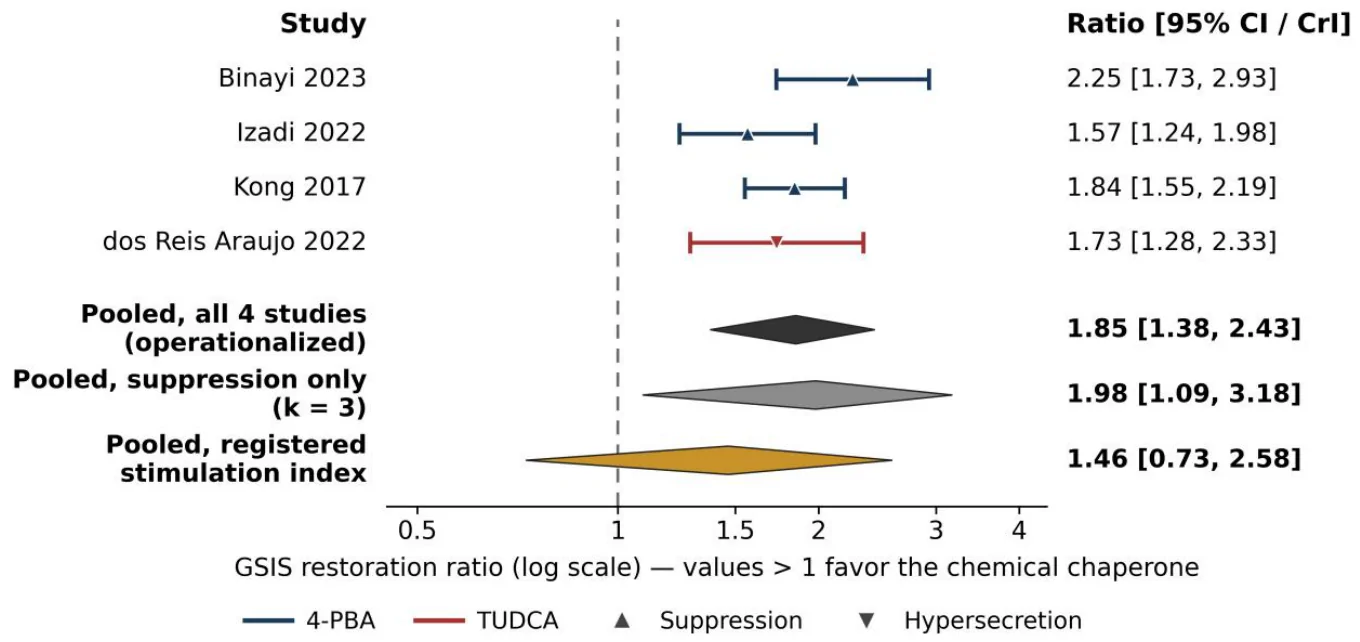
Forest plot of the per-study GSIS restoration ratio (chemical chaperone vs. stressor, oriented toward the healthy-control value) with the pooled Bayesian random-effects estimates (diamonds). Horizontal bars denote 95% confidence intervals for individual studies, and the diamonds span 95% credible intervals; blue = 4-PBA, red = TUDCA. Upward triangles mark the three suppression models and the downward triangle marks the single hypersecretion model. The lower diamond is the leave-one-out estimate obtained after excluding the hypersecretion model, and the lowest diamond is the pooled estimate recomputed on the registered within-arm stimulation-index scale (Section 3.4) [17,18,19,20].

At the level of individual studies, the rescue was substantial and directionally consistent across the available models (Table 2; Figure 3). In the chronic HFD model, islet insulin output under 16.7 mM glucose was approximately 120 ± 14 ng/10 islets/90 min under the stressor and rose to approximately 270 ± 18 ng/10 islets/90 min after 4-PBA, a 2.25-fold restoration (95% CI 1.73 to 2.93) [17]. In the HFHFD model, secretion increased from approximately 115 ± 11 to 180 ± 13 ng/10 islets/90 min with 4-PBA (1.57-fold; 95% CI 1.24 to 1.98) [18]. In cholesterol-loaded βTC-6 cells, glucose-stimulated secretion recovered from approximately 95 ± 12 to 175 ± 15 ng/mg protein (standard deviations) after 4-PBA pre-treatment (1.84-fold; 95% CI 1.55 to 2.19) [19]. In the malnutrition-plus-HFD model, where the stressor instead produced hypersecretion, TUDCA normalized output at 11.1 mM glucose from approximately 0.90 ± 0.09 to 0.52 ± 0.06 ng/islet/h, a 1.73-fold movement toward the healthy-control value (95% CI 1.28 to 2.33) [20]. These per-study GSIS values are approximations digitized from the source figures and independently re-read by a second reader, whereas the chaperone doses, routes, schedules, and group sizes listed in Table 2 are taken directly from the primary reports.

**Figure 4:**
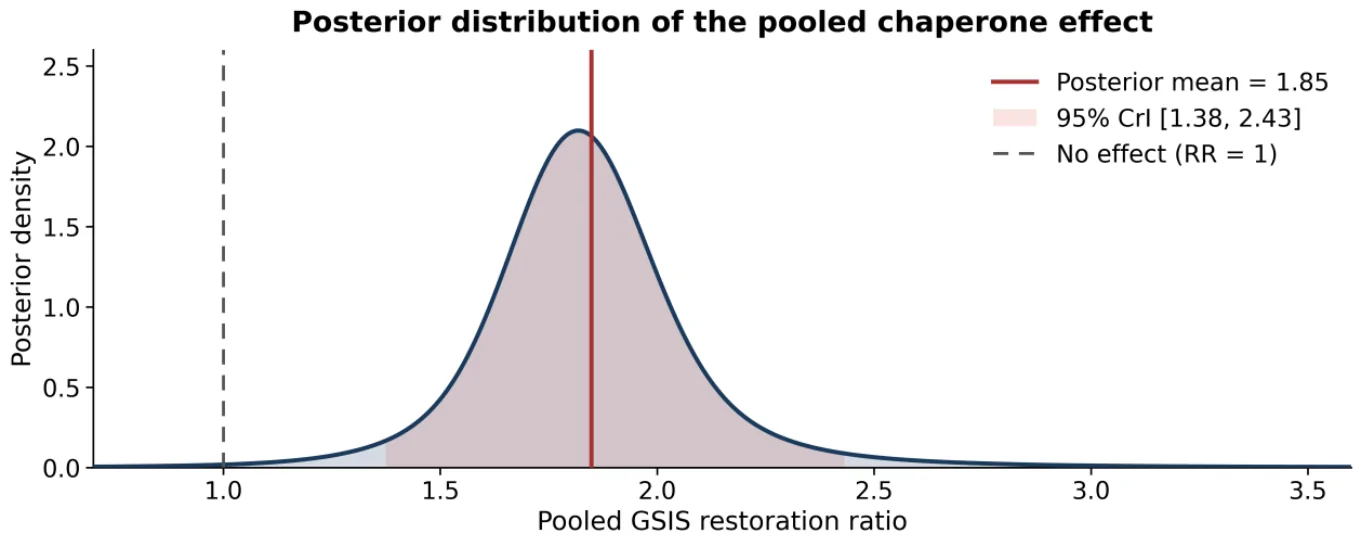
Posterior distribution of the pooled GSIS restoration ratio from the hierarchical Bayesian random-effects model. The posterior mean is 1.85 (95% CrI 1.38 to 2.43), with the entire credible mass above the no-effect line (RR = 1).

**Figure 5:**
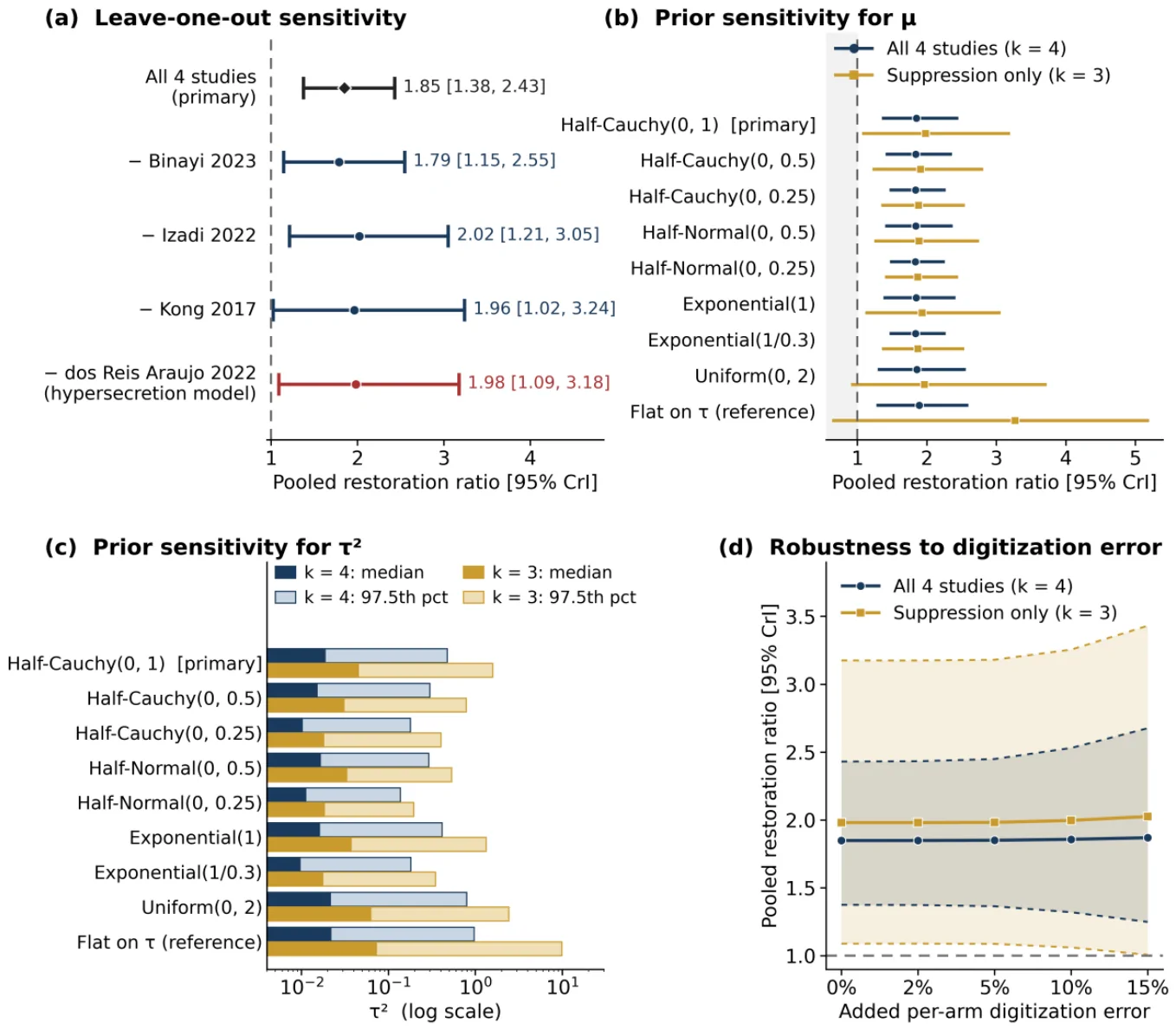
Sensitivity and robustness of the pooled GSIS restoration ratio. (**a**) Leave-one-out analysis; the red interval omits the single hypersecretion model. (**b**) Pooled effect under nine priors for the between-study standard deviation, for the four-study analysis (blue circles) and the suppression-only subset (gold squares). (**c**) Posterior median and 97.5th percentile of τ^2^ under the same priors, on a logarithmic scale; the pooled effect in (**b**) is stable while τ^2^ in (**c**) varies by almost an order of magnitude. (**d**) Pooled estimate and 95% credible interval as additional per-arm relative digitization error is added in quadrature to the within-study standard errors [17,18,19,20].

Pharmacological administration of 4-PBA or TUDCA moved secretory output toward the healthy-control phenotype in every included model. The hierarchical Bayesian model yielded a pooled posterior mean restoration ratio of μ = 1.85 (95% credible interval [CrI] 1.38 to 2.43), indicating a substantial positive effect of chemical chaperones on insulin secretion at stimulatory glucose across all experimental platforms (Table 2; Figure 3 and Figure 4). The entire credible interval lay above the no-effect value of 1, corresponding to a posterior probability of benefit of 0.996. The posterior for between-study variance was concentrated near zero (posterior median τ^2^ = 0.019; posterior mean 0.074; 95% CrI 0.000 to 0.475), and the prior-free descriptors were concordant on this scale (Cochran’s Q = 4.20, df = 3, *p* = 0.24; I^2^ = 28.6%); they are not concordant on the registered scale, as Section 3.4 reports. With only four studies, τ^2^ is weakly identified by the data and its posterior is strongly prior-dependent: across the eight alternative priors examined, the posterior median of τ^2^ ranged from 0.010 to 0.022 and its upper 97.5th percentile from 0.14 to 0.97 (Section 3.4; Figure 5c). The heterogeneity estimate is therefore reported as exploratory, and no claim of definitively low between-study heterogeneity is made. What the data do support is that the functional rescue was directionally consistent across the available models despite divergent biological platforms and stress protocols (Figure 4).

### 3.4. Sensitivity and Robustness Analyses

Removing the single hypersecretion model [20] left the direction and magnitude of the pooled effect essentially unchanged but reduced its precision: the posterior mean restoration ratio across the three suppression-only studies was 1.98 (95% CrI 1.09 to 3.18), with a posterior probability of benefit of 0.981 (Table 3; Figure 5a). Because that study is also the only TUDCA study, this analysis is simultaneously the 4-PBA-only subgroup analysis; the two questions—robustness to the secretory phenotype and freedom from a drug-specific mechanism—are answered by the same three studies and cannot be separated in this evidence base. Removing each of the other three studies in turn gave pooled estimates of 1.79 (95% CrI 1.15 to 2.55), 2.02 (1.21 to 3.05), and 1.96 (1.02 to 3.24); no single study drove the result, and every leave-one-out credible interval remained above unity. The hypersecretion model is not the source of instability: excluding it widened the credible interval by a factor of 2.0 and raised the posterior median of τ^2^ from 0.019 to 0.045, because with k = 3 the between-study variance becomes still less identifiable. The main quantitative conclusion is therefore framed around the full four-study set, with the suppression-only estimate reported alongside it for readers who regard the two phenotypes as non-exchangeable.

The pooled effect was insensitive to the prior placed on the between-study standard deviation; the heterogeneity parameter was not. Across the nine specifications examined (the primary Half-Cauchy(0, 1) and eight alternatives), the posterior mean restoration ratio in the four-study analysis varied only between 1.84 and 1.89, the lower limit of the credible interval between 1.30 and 1.49, and the posterior probability of benefit never fell below 0.99 (Table 3; Figure 5b). Over the same range of priors, the upper 97.5th percentile of τ^2^ varied sevenfold, from 0.14 to 0.97 (Figure 5c). This dissociation answers directly the question of whether a Half-Cauchy(0, 1) prior is defensible with k = 4: it is defensible for inference about μ, which is dominated by the likelihood, but no prior can render τ^2^ well identified from four studies, and the choice of prior should therefore be reported and varied rather than defended. In the suppression-only subset the pooled effect itself became prior-dependent, and under two of the nine priors the credible interval crossed unity: under Uniform(0, 2) the posterior mean was 1.97 (95% CrI 0.93 to 3.70; posterior probability of benefit 0.97), and under a flat reference prior on τ it rose to 3.27 (95% CrI 0.66 to 5.18; probability of benefit 0.95). The suppression-only estimate is therefore the more fragile of the two, which is a further reason to retain all four studies in the primary analysis and a caveat that attaches to the suppression-only estimate wherever it is quoted, including in Section 4.2.

Adding per-arm relative digitization error to the within-study standard errors had little effect. Inflating every arm by 2%, 5%, 10%, and 15% in quadrature moved the pooled restoration ratio from 1.849 to 1.849, 1.851, 1.858, and 1.869 respectively, and widened the credible interval from 1.38–2.43 to 1.25–2.68 at the most extreme setting, with the posterior probability of benefit remaining above 0.99 throughout (Table 3; Figure 5d). Because the two independent readings agreed to within a mean absolute relative difference of 2.8% (Section 2.3), the 10% and 15% settings are conservative by a wide margin. Two provenance analyses complete the set. Entering the dispersion of the study that reports standard deviations [19] as a standard error without converting it, as in the original submission, gives 1.85 (95% CrI 1.34 to 2.49); and retaining the originally submitted reading of the treated arm of [18] gives 1.87 (95% CrI 1.43 to 2.40). Neither correction alters the direction or the order of magnitude of the pooled estimate.

The registered and operationalized metrics are related by the exact identity given in Section 2.4, and their empirical divergence was quantified for all four studies by combining the stimulated-glucose values of Table 2 with the basal-glucose values reported in the same source figures. One caveat applies before the comparison is read: basal secretion was measured at 5.6 mM glucose in [17] and [18] and at 2.8 mM in [20], but at 0 mM in [19], so the stimulation index of the cholesterol model is not strictly the same quantity as that of the other three. In that model, the two metrics agreed closely, and basal secretion was explicitly unchanged by treatment (between-arm ratio 1.84 vs. stimulation-index ratio approximately 1.89) [19]. They diverged in the other three, and in opposite directions, because there the chaperone altered basal as well as stimulated secretion. In the chronic HFD model, 4-PBA raised basal output approximately 2.6-fold alongside stimulated output, so that the between-arm ratio (2.25) greatly exceeded the ratio of stimulation indices (approximately 0.88)—although the treated stimulation index (approximately 2.06) in fact moved toward the normal-diet control value (approximately 1.88) and away from the elevated value of the untreated stressor arm (approximately 2.35). In the HFHFD model, basal secretion fell slightly under treatment, so that the stimulation-index ratio (approximately 1.82) exceeded the between-arm ratio (1.57). In the hypersecretion model, both basal and stimulated output were lowered, giving a stimulation-index movement toward control of approximately 1.17 against a between-arm ratio of 1.73. The basal values underlying these calculations were read from the published figures and are approximations; they indicate direction and order of magnitude and are not offered as precise estimates.

Two conclusions follow. First, the operationalized metric measures restoration of absolute secretory capacity at stimulatory glucose, not restoration of fold glucose responsiveness; the two are interchangeable only when basal secretion is unaffected, and in these models, it frequently is not. The pooled estimate is therefore described throughout as restoration of glucose-stimulated insulin output rather than as restoration of the stimulation index. Second, the choice of metric is consequential, and the magnitude of that consequence can be stated exactly rather than bounded. Refitting the same model to the four per-study stimulation-index ratios—that is, pooling on the registered rather than the operationalized scale, with the basal ratio treated as known and the within-study standard errors unchanged—gives a pooled ratio of 1.46 (95% CrI 0.73 to 2.58; posterior probability of benefit 0.89), and in the suppression-only subset 1.85 (95% CrI 0.52 to 3.97; probability of benefit 0.85) (Table 3; Figure 3, lowest diamond). On that scale, the credible interval includes unity, and the studies are markedly heterogeneous: Cochran’s Q rises from 4.20 (df = 3, *p* = 0.24) to 28.01 (df = 3, *p* < 0.001) and I^2^ from 28.6% to 89.3%, because the chronic HFD model moves in the opposite direction to the other three on the stimulation-index scale (per-study ratios 0.88, 1.82, 1.89, and 1.17). Two features of this re-analysis should be borne in mind: the basal values on which it rests were read from the published figures and are approximations, and treating the basal ratio as known without error makes the interval reported here narrower, not wider, than a fully propagated one would be. The conclusion that the evidence supports is therefore restoration of absolute insulin output at stimulatory glucose; restoration of fold glucose responsiveness is not established by these four studies. The prior-free heterogeneity descriptors quoted in Section 3.3 are likewise properties of the operationalized scale and should not be read as properties of the evidence independent of it. The interpretive consequences are developed in Section 4.3 and Section 4.6.

### 3.5. Secondary Outcome: Expression and Mitigation of BiP and CHOP

The principal mechanism underlying preservation of insulin secretion was direct suppression of terminal ER-stress signaling [17]. Western blot analyses demonstrated that glucolipotoxicity induced a marked upregulation of BiP/GRP78, ATF4, and the terminal pro-apoptotic transcription factor CHOP [19]. Co-treatment with 4-PBA or TUDCA significantly blunted this response [17]. In the pancreas of HFD-fed rats, 4-PBA decreased BiP and CHOP levels and restored the ER-membrane glycoprotein WFS1 [17]. Three of the four included studies therefore provide direct quantification of ER-stress markers, and every statement made here about marker expression rests on those three. In the malnutrition-plus-HFD model, BiP and CHOP were not measured; instead, TUDCA normalized the glutamate-dehydrogenase-driven islet insulin hypersecretion and improved peripheral insulin sensitivity [20]. That study was retained because eligibility was defined by the population, the intervention, the comparator, and the availability of insulin-secretion data, and not by the availability of ER-stress readouts (Section 2.2 and Section 2.6). It contributes to the primary functional synthesis and to the description of downstream metabolic consequences; it contributes to no pooled ER-stress estimate, because none was computed. The hierarchical mechanism framework is thus supported by direct measurement in every study that contributes to it, while the functional synthesis draws on all four.

### 3.6. Molecular Mechanism of 4-PBA: Structural Scaffolding and WFS1 Stabilization

4-PBA acts predominantly by binding hydrophobic regions of unfolded or partially folded proteins in the ER lumen, preventing nonspecific aggregation and facilitating structural maturation and transit through the secretory pathway [19]. Under overnutrition, the rapid influx of nascent proinsulin exceeds the folding capacity of BiP [18]; 4-PBA reduces the thermodynamic energy required for proinsulin folding, alleviating ER congestion [19]. Crucially, this reduction in luminal stress is accompanied by stabilization of the ER-membrane glycoprotein Wolfram syndrome 1 (WFS1) [17]. WFS1 is a substrate of the E3 ubiquitin ligase Smurf1, which targets it for proteasomal degradation at the ER, and Smurf1 itself is regulated by ER stress [21]. That mechanism was characterized in heterologous cell systems and was not tested with chemical chaperones; the inference that 4-PBA limits Smurf1-mediated WFS1 loss by preventing UPR hyperactivation is therefore ours and is offered as a mechanistic hypothesis consistent with the WFS1 stabilization observed under 4-PBA [17] rather than as a demonstrated step. Because WFS1 maintains ER calcium homeostasis, its stabilization prevents excessive calcium leaks into the cytosol and preserves the calcium-regulated exocytotic machinery [17].

### 3.7. Molecular Mechanism of TUDCA: Receptor-Mediated Signaling

In contrast to 4-PBA, TUDCA is a hydrophilic bile-acid conjugate with a dual mechanism, combining physical chaperone activity in the ER with cell-surface receptor-mediated signaling [22]. Pharmacological evidence indicates that TUDCA engages the G protein-coupled bile-acid receptor TGR5 on the beta-cell membrane: its secretory effect is abolished by inhibition of Gαs and is reproduced by the selective TGR5 agonist INT-777 [22], and TUDCA promotes stimulus–secretion coupling through an adenylyl cyclase/cyclic AMP/protein kinase A (PKA)-dependent pathway in mouse and human islets alongside established TGR5 agonists [23]. Genetic confirmation in TGR5-null beta-cells has not been reported, and TUDCA also signals through sphingosine-1-phosphate receptor 2 and α5β1-integrin in this cell type [24,25]; TGR5 should therefore be regarded as a well-supported but not exclusive route. Elevated cyclic AMP activates PKA, which phosphorylates CREB [22] and potentiates the late, Ca^2+^-dependent steps of insulin-granule exocytosis (docking, priming, and SNARE-mediated fusion) [22,26]. This potentiation is glucose-dependent: at basal glucose (2.8 mM), TUDCA does not stimulate secretion, thereby avoiding hypoglycemia; at stimulatory glucose (11.1–22.2 mM), it dose-dependently potentiates secretion and shifts the glucose-response curve leftward [22].

### 3.8. Downstream Systemic and Peripheral Metabolic Repercussions

The benefits of chemical-chaperone therapy extend beyond the pancreas to systemic lipid and glucose metabolism [2]. In streptozotocin-induced diabetic models, TUDCA (300 mg/kg i.p. daily) reduced blood glucose, HbA1c, and HOMA-IR, increased serum insulin and GLP-1, and decreased the serum levels and activity of ceramide synthase, thereby limiting the accumulation of lipotoxic ceramides [27]. In prediabetic-aged mice, TUDCA attenuated hyperinsulinemia by restoring hepatic insulin clearance: aging downregulates hepatic insulin-degrading enzyme (IDE), and TUDCA upregulated hepatic IDE through S1PR2/Akt signaling, normalizing circulating insulin and restoring peripheral insulin sensitivity [2]. This normalization was accompanied by reduced visceral adiposity, lower hepatocyte triglyceride content, increased whole-body energy expenditure, and reversal of age-related cognitive decline [2]. In offspring exposed to an HFHFD from birth to young adulthood, 4-PBA improved insulin resistance (lower HOMA-IR, higher QUICKI), reduced pancreatic and hypothalamic malondialdehyde, downregulated BiP and CHOP, and lowered plasma leptin, although the diet-induced fall in HDL and rise in LDL persisted [18].

## 4. Discussion

The integrated preclinical evidence indicates that chemical chaperones are a promising strategy for arresting beta-cell functional decline in T2DM. Rather than merely delaying cell death, TUDCA and 4-PBA appear to preserve exocytotic identity by maintaining the ER folding environment during chronic glucolipotoxic stress.

The principal finding of this synthesis is quantitative: across four mechanistically distinct preclinical models, co-administration of TUDCA or 4-PBA produced a large and directionally consistent movement of insulin secretion at stimulatory glucose toward the healthy-control phenotype, corresponding to a pooled Bayesian posterior mean of 1.85 (95% CrI 1.38 to 2.43) on the restoration-ratio scale (Figure 3 and Figure 4). Because the entire credible interval lies above the null, the posterior probability that chemical chaperones improve secretory output under glucolipotoxic stress is 0.996, and this conclusion survived every leave-one-out and prior-sensitivity analysis performed on that scale, though not the change in scale itself (Section 3.4). The posterior for between-study variance was concentrated near zero (posterior median τ^2^ = 0.019; I^2^ = 28.6%), but with four studies that parameter is weakly identified and strongly prior-dependent, so it is reported as exploratory rather than as evidence of definitively low heterogeneity. What can be said is that despite divergent stressors (chronic high-fat feeding, high-fat/high-fructose feeding, cholesterol loading, and protein restriction followed by high-fat feeding) and platforms (in vivo and ex vivo islets and immortalized beta-cell lines), the secretory rescue was directionally consistent across the available models. This convergence is compatible with a shared upstream mechanism—relief of ER proteostatic stress—rather than with model-specific idiosyncrasies, and it reinforces the biological plausibility of the pooled estimate; with k = 4 it does not, however, constitute a demonstration of exchangeability. That qualification bears on what the pooled parameter estimates. In a hierarchical model, μ is the mean of a population of exchangeable study-level effects; here that population is a small and heterogeneous set of preclinical platforms, so μ is best read as the average movement toward the healthy-control phenotype across the models actually available, and not as a transportable effect in any one of them. Section 4.2 develops the sense in which the two secretory phenotypes are and are not exchangeable.

Two features distinguish the present work. First, rather than cataloging chaperone effects qualitatively, it integrates them within a single hierarchical Bayesian framework that propagates both within- and between-study uncertainty, yielding an interpretable probabilistic estimate well suited to the small, noisy datasets characteristic of basic beta-cell research. Second, it reconciles two mechanistically separable modes of action—the purely physical scaffolding of 4-PBA, which prevents proinsulin aggregation and is accompanied by preservation of WFS1 [17], a protein whose turnover at the ER is controlled by Smurf1 [21], and the dual chaperone-plus-receptor activity of TUDCA, which additionally engages TGR5/cAMP/PKA signaling to potentiate the SNARE-dependent late steps of granule exocytosis [22,26]—within a single account of how ER proteostasis is coupled to secretory identity. The translational relevance is heightened by the clinical maturity of both agents: 4-PBA (sodium phenylbutyrate) is an approved chemical chaperone for urea-cycle disorders with a well-characterized human safety profile [28], and TUDCA has been administered safely to insulin-resistant adults at a dose (1.75 g/day) closely matching the allometrically modeled effective dose [29].

### 4.1. Concordance with the Broader Preclinical Literature

Although only four studies met the registered analytic window and PICOS criteria for quantitative pooling, the direction and magnitude of the pooled effect are consistent with a broader preclinical literature that was not eligible for meta-analysis. 4-PBA restored palmitate-impaired GSIS in beta-cells [30], and TUDCA improved islet function by reducing ER stress [31]; chaperone treatment also ameliorated beta-cell dysfunction in islet-amyloid-polypeptide overexpression models [32] and preserved the UPR to protect against autoimmune (type 1) diabetes in NOD mice [33]. Early human data are likewise supportive: oral sodium phenylbutyrate partially alleviated lipid-induced beta-cell dysfunction in adults during clamp studies [34]. These reports were excluded from the quantitative synthesis because they fell outside the January 2016 analytic window or employed stressors (amyloid proteotoxicity, autoimmune cytokine attack) or designs (human clamp studies) that did not meet the registered PICOS criteria; they therefore inform the biological context and external consistency of the pooled estimate rather than the estimate itself.

### 4.2. Normalization of Impaired Versus Exaggerated Secretion

Three of the included models reproduce glucolipotoxic suppression of secretion, in which the chaperone raises a depressed secretory output [17,18,19], whereas the model of protein-restriction–programmed obesity reproduces insulin hypersecretion, in which TUDCA lowers an exaggerated output toward the level of its own dietary control [20]. Expressing all four on a common restoration-ratio scale oriented toward the healthy-control phenotype (Figure 1) asserts that both directions of adjustment represent the same underlying construct—normalization of secretory output—and not that they represent the same therapeutic situation. They do not. Suppressed secretion in the HFD, HFHFD, and cholesterol models corresponds to established beta-cell failure, in which the therapeutic goal is to recover lost secretory capacity. Hypersecretion in the malnutrition-plus-HFD model corresponds to the earlier, compensatory phase of the disease, in which the beta-cell is over-working to offset peripheral insulin resistance and the therapeutic goal is to relieve that workload; there the fall in insulin output produced by TUDCA follows from improved peripheral insulin sensitivity and reduced glutamate-dehydrogenase-driven secretion rather than from a direct secretagogue action [20]. ER-stress plausibly links the two phases, since the proteostatic burden that eventually exhausts the beta-cell is generated by the compensatory hypersecretion that precedes it; but the clinical populations implied are different, and a single pooled estimate should not be read as a single therapeutic indication. The leave-one-out analysis (Section 3.4) shows that the two phenotypes can be separated statistically without changing the qualitative conclusion: the suppression-only estimate is 1.98 (95% CrI 1.09 to 3.18), slightly larger than the four-study estimate and considerably less precise. Readers who regard the phenotypes as non-exchangeable may take that estimate as the relevant one for established beta-cell failure, with the caveat that it is the more prior-dependent of the two: under two of the nine priors examined its credible interval includes unity (Section 3.4).

### 4.3. What the Restoration Ratio Does and Does Not Measure

Two features of the effect-size metric constrain its interpretation. The first concerns the choice of glucose concentration. The pooled ratio is computed from secretion at stimulatory glucose and therefore measures restoration of absolute secretory capacity, not restoration of fold glucose responsiveness. As Section 3.4 shows, the two coincide when the chaperone leaves basal secretion untouched, as in the cholesterol model, and diverge otherwise; in the chronic HFD model the stressor depressed basal secretion proportionally more than stimulated secretion, so that the untreated stimulation index was actually higher than that of normal-diet controls, and 4-PBA restored absolute output at both glucose concentrations while returning the stimulation index toward the control value. This is substantive rather than technical: where glucolipotoxicity depresses secretion globally, the within-arm stimulation index is a poor index of beta-cell failure and the registered outcome would have understated the defect it was designed to capture. The between-arm metric was adopted for that reason; the consequence is that the pooled estimate must be described as restoration of glucose-stimulated insulin output and not as restoration of the stimulation index, and the text has been revised throughout to observe that distinction. The second feature concerns the mechanism of TUDCA. Because TUDCA potentiates the late, cyclic AMP/PKA-dependent steps of exocytosis in addition to acting as a chaperone (Section 3.7), it is legitimate to ask whether the pooled ratio reflects relief of ER stress or direct exocytotic potentiation. Three observations argue that it is not driven by the latter, although the first is structurally weaker than the other two. First, the three 4-PBA studies—and 4-PBA has no known bile-acid receptor activity—yield a pooled ratio of 1.98 (95% CrI 1.09 to 3.18), which overlaps the single TUDCA estimate of 1.73 (95% CI 1.28 to 2.33), so the effect is reproduced in the absence of a TGR5 component. This comparison must be read with care: in this evidence base, the single TUDCA study is also the single hypersecretion study, so drug, phenotype, and study are perfectly collinear, and no analysis can separate them; comparing a three-study pooled estimate with a single study is not a subgroup analysis, and no posterior for the difference is computed or claimed. Second, in the one TUDCA study, the drug decreased insulin secretion, whereas cyclic AMP/PKA-mediated potentiation acts in the opposite direction, so direct exocytotic potentiation would have attenuated rather than created the observed effect. Third, TUDCA in that study was given in vivo for 15 days, and secretion was measured ex vivo in isolated islets after withdrawal of the drug, a design that reports a persisting change in islet phenotype rather than the acute receptor-mediated potentiation described in perfusion experiments [22]. Confounding by acute TGR5/cyclic AMP signaling is therefore unlikely to account for the pooled effect—the second and third observations carry that argument, not the first—although it cannot be excluded as a contributor within the TUDCA arm itself, and the collinearity just described is a structural limitation of the available evidence rather than of the analysis.

### 4.4. Cellular Proteostasis and Systemic Metabolic Crosstalk

Organized by physiological domain, Table 4 shows that the two chaperones share a common glucose-normalizing action but differ in breadth. For glycemic control, TUDCA reversed fasting hyperglycemia, lowered HbA1c, and restored HOMA-IR and QUICKI toward control values [27], whereas 4-PBA lowered fasting glucose, reduced HOMA-IR, and raised QUICKI in the HFHFD-offspring model [18]. Both improved islet function; TUDCA restored glucose-dependent secretion and raised GLP-1 [27], and 4-PBA upregulated insulin mRNA and intracellular insulin content [18], consistent with the secretory rescue quantified in Section 3.3. Their lipid effects diverged in magnitude: TUDCA markedly lowered total cholesterol, triglycerides, LDL-C, and VLDL-C while raising HDL-C [27], whereas under 4-PBA, the diet-induced dyslipidemia persisted and was only partially corrected [18]. Both restored redox balance and dampened inflammatory and apoptotic signaling, with TUDCA suppressing TNF-α, IL-6, IL-1β, and PGE2 and blocking the intrinsic apoptotic cascade [27], and 4-PBA reducing CHOP-mediated apoptosis and preserving WFS1 [18], whose turnover at the ER is Smurf1-dependent [21], directly linking the systemic readouts to the ER-proteostatic mechanism of Section 3.5 and Section 3.6. Collectively, these data are compatible with 4-PBA acting as a predominantly proteostatic, β-oxidation-promoting agent and TUDCA as a broader metabolic and neuroprotective modulator, while both converge on improved glycemic control. The two agents were never compared within a single experiment; however, this contrast rests on an indirect comparison between studies that differ in species, stressor, and design, and is offered as such.

### 4.5. Allometric Calibration and Clinical Translation

To translate preclinical efficacy into clinical therapy, allometric dose calibration is required. In the single included rodent trial using TUDCA [20], and in the supporting rodent literature [2], the intraperitoneal TUDCA dose was 300 mg/kg. Using the body-surface-area conversion to the human-equivalent dose (HED), HED = animal dose × (K_m_, animal/K_m_, human), with K_m_ = 3 for the mouse and 37 for the adult human [2], a 300 mg/kg mouse dose corresponds to a human-equivalent dose of approximately 24.3 mg/kg/day, or about 1.7 g/day for a 70 kg adult. This estimate is closely consistent with the dose already shown to be safe and metabolically active in humans: obese, insulin-resistant adults received 1.75 g/day of TUDCA for four weeks without adverse events and with improved hepatic and muscle insulin sensitivity [29]. This convergence supports the translational plausibility of the modeled dose, although it modestly exceeds the 10–20 mg/kg/day range licensed for cholestatic liver disease [2]. Controlled clinical trials are required to confirm whether structural beta-cell preservation and restored insulin clearance observed in animals translate to patients with T2DM.

### 4.6. Limitations and Future Outlook

Several limitations temper these conclusions. First, the quantitative synthesis rests on only four eligible studies. Estimating a between-study variance from so few studies yields a heterogeneity estimate of very limited precision whose posterior is strongly prior-dependent, as the prior-sensitivity analysis demonstrates directly (Section 3.4; Figure 5c): the pooled effect varied by less than 4% across nine priors, whereas the upper limit of the τ^2^ interval varied sevenfold. The τ^2^ estimate is therefore exploratory, and no claim of definitively low heterogeneity is made. Second, the four eligible studies do not represent a single secretory phenotype, and expressing suppression and hypersecretion on a common restoration-ratio scale aggregates physiologically distinct therapeutic situations; the leave-one-out analysis excluding the hypersecretion model was performed for that reason (Section 3.4), and the interpretive distinction is drawn in Section 4.2. Third, the effect-size metric is a between-arm ratio at stimulatory glucose rather than the within-arm stimulation index named in the registered protocol, and BiP, WFS1, and cleaved caspase-3 are reported alongside the registered CHOP outcome; both deviations are declared in Section 2.6, and the empirical consequences of the first are quantified in Section 3.4 and discussed in Section 4.3. That first deviation is the most consequential limitation of the synthesis, and it should be stated plainly: when the same model is fitted on the registered stimulation-index scale, the pooled ratio falls to 1.46 with a credible interval that includes unity (95% CrI 0.73 to 2.58) and the studies become markedly heterogeneous (I^2^ = 89.3%). What these four studies support is restoration of absolute insulin output at stimulatory glucose; restoration of fold glucose responsiveness is not established by them, and the heterogeneity descriptors reported for the primary analysis are properties of the operationalized scale. Fourth, the primary effect sizes were derived from figure digitization because the insulin-secretion values were reported graphically rather than numerically in the source papers, and because the corresponding authors were not contacted to request the underlying numerical datasets; the synthesis therefore rests exclusively on the published full texts. Every digitized value was independently re-read from the source figure by a second reader, with a mean absolute relative difference of 2.8% between the two readings, and the effect of extraction error was bounded explicitly by variance inflation up to 15% (Section 3.4); residual extraction error nevertheless cannot be excluded, and no values were imputed where source data were unavailable. Obtaining the original datasets from the primary investigators would remove this source of uncertainty and is the appropriate remedy should the present estimate be carried forward. Fifth, the a priori restriction of the search to January 2016–May 2026 excluded concordant earlier studies (e.g., [30,31]) and studies employing non-glucolipotoxic stressors; these were retained only as qualitative context (Section 4.1) and did not contribute to the pooled estimate, but their exclusion narrows the evidence base relative to the full historical literature. Sixth, none of the four source reports documents blinding of outcome assessors during the insulin-secretion assays or the Western blot densitometry, so detection bias is unclear throughout. This bears on clinical generalizability in a specific way. Unblinded assessment of a continuous outcome read from an immunoassay plate, or a densitometric scan, is expected to bias effect estimates away from the null, which is precisely why blinding constitutes a separate domain of the SYRCLE instrument [10]. That expectation is an argument from the design of the instrument rather than an effect size estimated for this literature, and we report it as such; the pooled ratio of 1.85 should therefore be read as an upper bound on the true preclinical effect rather than as an unbiased estimate of it, and as a weaker guide still to any effect obtainable in patients, in whom the comparator would be standard care rather than a vehicle-injected littermate and the endpoint would be glycemic control rather than ex vivo islet output. The defensible inference is that the direction of effect is well supported while its magnitude is not transportable; confirmatory human trials should be powered on clinical endpoints rather than on an allometric extrapolation of this ratio. Seventh, with fewer than ten studies, formal small-study and publication-bias diagnostics were not informative, so the possibility that null preclinical results remain unpublished cannot be assessed and is a further reason to treat the pooled magnitude as an upper bound. The principal evidence gap remains the absence of controlled human trials translating these preclinical findings into human islet physiology; future work should prioritize trials in prediabetes or early-stage T2DM and should report insulin secretion at both basal and stimulatory glucose, so that secretory capacity and glucose responsiveness can be distinguished rather than conflated. Eighth, two features of the evidence base cannot be repaired by analysis: the single TUDCA study is also the single hypersecretion study, so drug and phenotype are perfectly collinear (Section 4.3), and the primary values of two studies were corrected during revision after re-reading the source figures (Table 2 footnotes; Table 3, provenance rows), which indicates the precision attainable when effect sizes must be recovered from published graphs.

## 5. Conclusions

Pharmacological administration of the chemical chaperones TUDCA and 4-PBA preserved pancreatic beta-cell secretory function under severe glucolipotoxic stress in every preclinical model available for synthesis. By alleviating ER stress, these low-molecular-weight compounds appear to limit terminal UPR activation, downregulate pro-apoptotic CHOP and the chaperone BiP, protect the ER-membrane glycoprotein WFS1, and restore insulin secretion at stimulatory glucose, with a pooled Bayesian restoration ratio of 1.85 (95% CrI 1.38 to 2.43) that was directionally consistent across all four available models and robust to leave-one-out, prior, and extraction-error sensitivity analyses. Two qualifications attach to that figure. The evidence base is four preclinical studies with unclear detection bias, so its magnitude should be regarded as an upper bound rather than as an unbiased estimate; and it is computed on a between-arm metric that departs from the registered within-arm stimulation index, on which scale the pooled interval includes unity. The claim the evidence supports is restoration of absolute glucose-stimulated insulin output, not of fold glucose responsiveness. Combined with TUDCA’s capacity to restore hepatic insulin-degrading-enzyme (IDE)-mediated insulin clearance, reduce systemic lipotoxicity, and improve cognition, these findings position chemical chaperones as promising disease-modifying candidates to halt the cellular progression of T2DM, pending confirmation in controlled human trials [2].

## Figures and Tables

**Table 1:** Characteristics and SYRCLE risk-of-bias assessment of the four included preclinical studies. Direct quantification of endoplasmic-reticulum-stress markers was a secondary outcome, not an eligibility criterion; the single study that did not report CHOP or BiP [20] contributes to the primary insulin-secretion synthesis and to the mechanistic narrative and contributes to no pooled ER-stress estimate (Section 2.2 and Section 3.5).

Study (Year)	Design & Model	Metabolic Stressor	Intervention	Primary Outcome (GSIS)/Secondary (ER Stress)	SYRCLE Risk of Bias
Binayi et al. (2023) [17]	In vivo & ex vivo (male Wistar rats; isolated islets)	High-fat diet (HFD), 20 weeks (chronic lipotoxicity)	4-PBA, 50 mg/kg, i.p., twice daily × 3 d	HFD reduced GSIS and total insulin content; 4-PBA restored GSIS. HFD raised pancreatic CHOP and BiP; 4-PBA mitigated both.	Low/unclear (randomized allocation; detection blinding unclear; reporting unclear—primary values reported graphically only)
Izadi et al. (2022) [18]	In vivo & ex vivo (male rat offspring; isolated islets)	High-fat/high-fructose diet (HFHFD) (chronic glucolipotoxicity)	4-PBA, 50 mg/kg, i.p., twice daily × 10 d	4-PBA increased insulin mRNA and restored GSIS; reduced HFHFD-induced pancreatic CHOP and BiP.	Low/unclear (low attrition; detection blinding unclear; reporting unclear—primary values reported graphically only)
Kong et al. (2017) [19]	In vitro (rat INS-1 and mouse βTC-6 insulinoma cells; insulin secretion measured in βTC-6)	Cholesterol loading, 5 mM × 6 h (lipotoxicity)	4-PBA, 1 mM, pre-treatment × 24 h	4-PBA reversed the cholesterol-induced fall in insulin secretion in βTC-6 cells and prevented injury; reversed CHOP induction and attenuated cleaved caspase-3 in both lines.	Low/unclear (robust internal vehicle controls; detection blinding unclear; reporting unclear—primary values reported graphically only)
dos Reis Araujo et al. (2022) [20]	In vivo & ex vivo (C57BL/6 mice; isolated islets)	Protein restriction (6%) followed by HFD (35% fat)	TUDCA, 300 mg/kg, i.p., during the last 15 d	HFD induced islet insulin hypersecretion at 11.1 mM glucose; TUDCA normalized secretion and improved glucose tolerance. CHOP/BiP not assessed (not an eligibility criterion); TUDCA lowered islet GDH and raised IRβ/GLUT4.	Low/unclear (strict biological controls; low-performance bias; detection blinding unclear; reporting unclear—primary values reported graphically only)

**Table 2:** Chemical-chaperone dosing regimens and GSIS outcomes extracted from the four included studies, with the per-study restoration ratio entering the Bayesian meta-analysis.

Study (Year)	Chaperone, Dose, Route, Schedule	Model & Metabolic Stressor	GSIS, Stressor Only (Mean ± SEM or SD; *n*)	GSIS, + Chaperone (Mean ± SEM or SD; *n*)	Restoration Ratio [95% CI]
Binayi et al. (2023) [17] ▲	4-PBA, 50 mg/kg, i.p., twice daily × 3 d (after 20-wk HFD)	Male Wistar rat islets, ex vivo; HFD, 20 wk	120 ± 14 (*n* = 4) †	270 ± 18 (*n* = 4) †	2.25 [1.73, 2.93]
Izadi et al. (2022) [18] ▲	4-PBA, 50 mg/kg, i.p., twice daily × 10 d	Male rat offspring islets, ex vivo; HFHFD, birth→PND 66	115 ± 11 (*n* = 6) †	180 ± 13 (*n* = 6) †§	1.57 [1.24, 1.98]
Kong et al. (2017) [19] ▲	4-PBA, 1 mM, pre-treatment × 24 h	βTC-6 cells (insulin secretion) and INS-1 cells, in vitro; cholesterol 5 mM × 6 h	95 ± 12 (SD; *n* = 3–5) †‡	175 ± 15 (SD; *n* = 3–5) †‡	1.84 [1.55, 2.19]
dos Reis Araujo et al. (2022) [20] ▼	TUDCA, 300 mg/kg, i.p., during the last 15 d	C57BL/6 mouse islets, ex vivo; 6% protein → HFD (35% fat)	0.90 ± 0.09 (*n* = 7) †	0.52 ± 0.06 (*n* = 6) †	1.73 [1.28, 2.33]
Pooled (Bayesian random-effects)	4-PBA and TUDCA	4 studies; 4 stress models	—	—	1.85 [1.38, 2.43]

Dosing regimens, group sizes (*n*), glucose concentrations, stressor protocols, and the dispersion measure reported by each source are text-reported in the primary publications and are verifiable. ▲ denotes a suppression phenotype (the metabolic stressor lowered secretion) and ▼ a hypersecretion phenotype (the stressor raised secretion); in both cases the restoration ratio is oriented so that values above unity denote movement of secretory output toward the healthy-control phenotype (Figure 1). GSIS values (†) were digitized from the source figures (ref. [17], Figure 5B; ref. [18], Figure 8A; ref. [19], Figure 4K; ref. [20], Figure 3K) and are approximations; the corresponding basal-glucose values used in the sensitivity analysis of Section 3.4 are reported in the same figures (ref. [17], Figure 5A; ref. [18], Figure 8A, left block; ref. [19], Figure 4K, “BIS”; ref. [20], Figure 3K, 2.8 mM block). GSIS was assessed at high-glucose stimulation (16.7 mM for Binayi and Izadi; 25 mM for Kong; 11.1 mM for dos Reis Araujo). Units: ng/10 islets/90 min (Binayi, Izadi); ng/mg protein (Kong); ng/islet/h (dos Reis Araujo). ‡ Kong et al. report dispersion as the standard deviation of three to five independent experiments; it was converted to the standard error with the conservative *n* = 3, and the effect of entering it unconverted is reported in Table 3. § The treated-arm value of Izadi et al. was corrected during revision from 190 to 180 ng/10 islets/90 min after independent re-reading of Figure 8A of that report; the effect of retaining the originally submitted reading is also reported in Table 3. The pooled estimate is the posterior mean (95% CrI) from the hierarchical Bayesian random-effects model.

**Table 4:** Physiological, metabolic, and histological parameters regulated by TUDCA and 4-PBA across the included and supporting preclinical models. Every entry is drawn from the two reports cited for it in Section 3.8: the TUDCA column from [27] and the 4-PBA column from [18]. Abbreviations: HbA1c, glycated hemoglobin; HOMA-IR, homeostatic model assessment of insulin resistance; QUICKI, quantitative insulin-sensitivity check index; LDL-C, low-density-lipoprotein cholesterol; VLDL-C, very-low-density-lipoprotein cholesterol; HDL-C, high-density-lipoprotein cholesterol; TNF-α, tumor necrosis factor α; IL, interleukin; PGE2, prostaglandin E2; MDA, malondialdehyde; CAT, catalase; GSH, reduced glutathione; NO, nitric oxide; GDH, glutamate dehydrogenase; IRβ, insulin-receptor β subunit; GLUT4, glucose transporter 4; S1PR2, sphingosine-1-phosphate receptor 2. The two chaperones were not compared directly in any single experiment; the contrast drawn in Section 4.4 is therefore indirect.

Parameter	Untreated Metabolic Stress	Effect of TUDCA	Effect of 4-PBA
Glycemia & HOMA-IR	Fasting hyperglycemia, insulin resistance, elevated HOMA-IR, reduced QUICKI	Reverses hyperglycemia, lowers HbA1c, restores HOMA-IR and QUICKI	Lowers fasting glucose, reduces HOMA-IR, restores insulin-sensitivity index
Islet function & GSIS	Decline in GSIS at stimulatory glucose; depleted insulin content	Restores glucose-dependent secretion, raises GLP-1, normalizes insulinemia	Upregulates insulin mRNA, increases GSIS, restores intracellular insulin
Lipid profile	Elevated TC, TG, LDL-C; decreased HDL-C	Lowers TC, TG, LDL-C, VLDL-C; raises HDL-C	Partially mitigates dyslipidemia
Hepatic steatosis	Ectopic lipid, increased liver TG and cholesterol	Attenuates steatosis via S1PR2/Akt and AMPK	Reduces hepatocellular lipid, promotes β-oxidation
Redox status	Depleted CAT and GSH; elevated MDA and NO	Restores SOD, CAT, GSH; reduces MDA, NO, iNOS	Restores catalase and GSH; reduces pancreatic and hypothalamic MDA
Inflammation	Elevated TNF-α, IL-6, IL-1β, PGE2	Suppresses TNF-α, IL-6, IL-1β, PGE2	Decreases local and systemic cytokines
Apoptosis	Upregulated p53, Bax/Bcl-2, caspase-3	Downregulates p53 and caspase-3; blocks intrinsic apoptosis	Decreases CHOP-mediated apoptosis; prevents Smurf1-WFS1 degradation
Energy & cognition	Decreased energy expenditure; cognitive decline	Increases energy expenditure; restores novel-object memory	Stimulates β-oxidation; corrects hypothalamic insulin/leptin programming

## Data Availability

All data analyzed in this systematic review and meta-analysis derive from the published full texts of the four included reports and are reported in Table 1, Table 2 and Table 3 and in the Supplementary Materials. No unpublished data were obtained from the original investigators. The extracted per-study values, the annotated Bayesian model code, and the scripts that reproduce every figure and every sensitivity analysis are provided as Supplementary Materials and are additionally available from the corresponding author on reasonable request. No analysis reported here depends on data that are not included with this submission.

## Supplementary Materials

The following supporting information can be downloaded at: https://www.mdpi.com/article/10.3390/ijms27167267/s1.
